# Unconventional but effective: breaking through IBS-D clinical practice guidelines – correspondence

**DOI:** 10.1097/MS9.0000000000000338

**Published:** 2023-03-27

**Authors:** Omer Ahmed Shaikh, Gulrukh Shaikh, Rameel Muhammad Aftab, Haisum Baktashi, Irfan Ullah, Muhammad Sohaib Asghar

**Affiliations:** a Department of Medicine, Ziauddin University; b Department of Medicine, Liaquat National Hospital and Medical College, Karachi; c Kabir Medical College, Gandhara University, Peshawar, Pakistan; d Division of Nephrology and Hypertension, Mayo Clinic, Rochester, Minnesota, USA

**Keywords:** constipation, diarrhea, dyspepsia, gastroenterology, IBS

## Abstract

Irritable Bowel Syndrome (IBS) is a chronic, one of the commonest and persistent gastrointestinal (GI) disorder. Previously, the management plan for IBS-D included enhancing awareness; first line treatment included an increased dietary fiber intake, opioids for diarrhea and antispasmodics for pain management. A recent treatment guideline by the American Gastroenterology Association (AGA) suggests a modified approach to treating patients with IBS-D. Eight drug recommendations were made, and a set of instructions on when to employ which medication was devised. With the incorporation of these structured guidelines, a more tailored and focused approach to IBS management may become plausible.

Irritable bowel syndrome (IBS) is a chronic persistent gastrointestinal (GI) disorder affecting a population of ∼9–23% worldwide^1^. IBS is one of commonest GI diseases that prevails among the masses. It has a multifaceted origin, with symptoms coinciding with those of celiac disease and inflammatory bowel disease. Food sensitivity, carbohydrate malabsorption, modified GI motility, brain–gut interactions, and bacterial spurge are all risk factors associated with IBS^2^. There may be an array of symptoms, which may vary in nature. The most typical symptom that the patient might complain about is abdominal pain. However, it can also manifest as a constellation of other symptoms, including abdominal discomfort, bloating, diarrhea, constipation, and/or non-GI symptoms such as fatigue, stress, and anxiety^1^,^3^. There is a very potent association between depression, stress, and anxiety with IBS. Distressing experiences such as emotional and sexual abuse have also been believed to be the causative factor in the initiation of IBS. Patients with severe IBS may also have an accompanying psychiatric illness such as an eating disorder, depression, or somatoform disorder^3^.

The diagnosis of IBS is done on the basis of clinical symptoms in accordance with the Rome III criteria, which requires the patient to have been in abdominal discomfort for a minimum of 3 days over a time period of 3 months, with symptoms beginning at least 6 months before the onset of pain/discomfort. IBS is branched into four subtypes based on the Rome III criteria for diagnosis: IBS-C (constipation), IBS-D (diarrhea), IBS-M (mixed), and IBS-U (unsubtyped). There is also the Rome IV criteria, which is a modified approach to Rome III, adding that the repetitive abdominal pain must appear on an average of a minimum of 1 day throughout the week in the previous 3 months and may be in association with defecation, that is either increasing or remaining unchanged by defecating^4^.

Previously, the management plan for IBS-D included enhancing the patient’s knowledge regarding the disease so that it could be managed appropriately; first-line treatment included increased dietary fiber intake, opioids for diarrhea, and antispasmodics for pain management^5^.

A recent treatment guideline by the American Gastroenterology Association (AGA) suggests a modified approach to treating patients with IBS-D^6^. This is the first time a structured approach to treating IBS-D has been assumed. The guideline instructs with clear indications when to adhere to previously used U.S. Food and Drug Administration approved drugs and when to resort to over-the-counter medications. The guideline was formed using the Grading of Recommendations Assessment, Development and Evaluation (GRADE) framework. Eight drug recommendations were made, and a set of instructions on when to employ which medication was devised. Rifaximin, eluxadoline, loperamide, alosetron, tricyclic antidepressants, antispasmodics, and selective serotonin reuptake inhibitors were among the medications recommended.

Previously, there was no targeted plan for the treatment of IBS; it was managed by employing a holistic approach and using a variety of methods, including over-the-counter and herbal medications, psychotherapy, amendments to lifestyle, dietary adjustments, probiotics, and medication that targets the gut motility, sensation, and intraluminal milieu of patients with IBS-D.

With the incorporation of these structured guidelines, a more tailored and focused approach to IBS management may become plausible. Medical practitioners will now have a definite route to maneuver in order to treat specific patients who have a particular set of symptoms.

A separate guideline for the management of IBS-C has also been introduced, where a set of nine medications have been approved for the treatment of IBS-C^7^. However, a separate guideline for the management of IBS-M is yet to be introduced.

## Ethical approval

None.

## Sources of funding

No financial support was acquired for this article.

## Conflicts of interest disclosure

The authors declare no conflicts of interest.

## Provenance and peer review

Externally peer-reviewed, not commissioned.
